# Molecular detection of *Borrelia burgdorferi* sensu lato in questing ticks: One year of sampling in Bologna (Northern Italy)

**DOI:** 10.1016/j.onehlt.2026.101478

**Published:** 2026-06-16

**Authors:** Alessandra Mistral De Pascali, Laura Dionisi, Bianca Vandelli, Carola Valenti, Sara Rigamonti, Emanuela Olivieri, Nadia Vicari, Martina Brandolini, Ludovica Ingletto, Giulia Gatti, Claudia Colosimo, Massimiliano Guerra, Giorgio Dirani, Silvia Zannoli, Laura Grumiro, Sofia Montanari, Anna Marzucco, Monica Cricca, Roberto Cazzolla Gatti, Alessandra Scagliarini, Vittorio Sambri

**Affiliations:** a Unit of Microbiology, The Greater Romagna Area Hub Laboratory, 47522 Cesena, Italy; b Department of Medical and Surgical Sciences (DIMEC), University of Bologna, 40138 Bologna, Italy; c Department of Biological, Geological and Environmental Sciences (BIGEA), University of Bologna, 40126 Bologna, Italy; d Istituto Zooprofilattico Sperimentale della Lombardia e dell'Emilia-Romagna, Pavia, Italy

**Keywords:** Ticks, *Borrelia*, Seasonality, Prevalence

## Abstract

*Borrelia burgdorferi* sensu lato (s.l.) includes several genospecies responsible for Lyme borreliosis (LB), a major zoonotic disease transmitted by *Ixodes* spp. ticks. In Western Europe, LB incidence has risen in recent decades, with documented expansion into previously non-endemic regions. A cross-sectional study was conducted in peri-urban areas of Bologna, Emilia-Romagna (north-eastern Italy), from February to November 2024 to assess the presence of *B. burgdorferi* s.l. in questing ticks using real-time PCR. A total of 887 ticks were collected by dragging across 24 sites, including urban parks, school gardens, and forested areas. The 887 ticks were pooled into 130 samples and screened for *B. burgdorferi* s.l., resulting in an overall estimated prevalence of 10% in nymphs (95% CI 6.6–13.5) and 8% in adults (95% CI 1.4–22.7). This study provides the first prevalence estimate of *B. burgdorferi* s.l. in ticks within the municipality of Bologna. Pathogen detection in highly frequented peri-urban sites indicates a concrete exposure risk for human and animals. These findings support the need for strengthen local surveillance and targeted prevention strategies in line with One Health principles.

## Highlights

• *Borrelia burgdorferi,* Lyme disease agent, transmitted by *Ixodes* ticks, thrives in humid conditions
• The study was conducted in Emilia Romagna (North-eastern Italy) between February and November 2024
• 887 ticks were tested by PCR for the identification of *Borrelia burgdorferi* s.l.
• Real-time PCR testing of the 887 ticks, grouped into 130 samples, revealed an overall prevalence of 10% in nymphs (95% CI 6.6–13.5), and 8% in adults (95% CI 1.4–22.7).
  Study shows *B. burgdorferi* s.l. high prevalence in ticks, urging year-round surveillance and new plans of monitoring

## Graphical abstract

**Figure:**
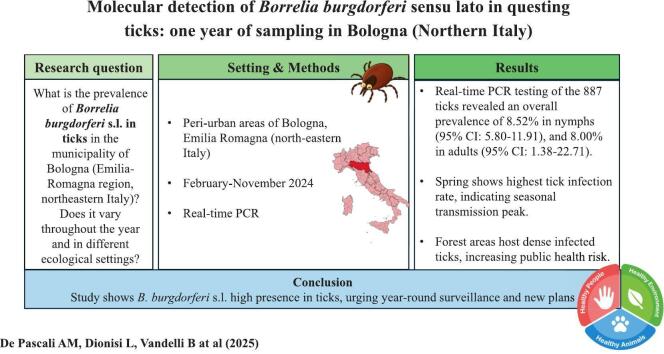

## 1 Introduction

Lyme borreliosis (LB) is a zoonotic disease caused by certain members of the *Borrelia burgdorferi* sensu lato (s.l.) complex. In Europe, *B. burgdorferi* s.l. is predominantly transmitted to vertebrate hosts by ticks of the *Ixodes ricinus* complex, which maintain the pathogen through enzootic cycles involving mammals, birds, and reptiles [1]. *Borrelia burgdorferi* s.l. is transmitted to ticks through attachment and feeding on an infected host, a process known as systemic transmission [2], and is maintained across developmental stages via transstadial transmission. Relative humidity (above 80–85%) is a key factor influencing *Ixodes ricinus* survival and activity, as immature stages are highly sensitive to desiccation. Microclimatic parameters play an important role in shaping tick behaviour, survival, and pathogen transmission dynamics [3]. In northern and central Europe, nymphs and adults are active from March to November, with activity peaking in April and May. Seasonal patterns are influenced by temperature and moisture, with warm, dry periods reducing activity, while mild winters favouring extended season persistence [2], [4], [5].

*Borrelia burgdorferi* s.l. comprises at least 20 named *Borrelia* species, including *B. burgdorferi* sensu stricto (s.s.), *B. afzelii, B. garinii, B. bavariensis* and *B. spielmanii*, which are responsible for human LB in Europe (Cardenas-de La Garza et al., 2019). Erythema migrans is a common symptom that may resolve without antibiotic treatment. However, the pathogen can disseminate, causing serious multisystem disorders affecting skin, nervous system, heart, and muscles [6], such as Lyme neuroborreliosis, Lyme carditis, Lyme arthritis, and acrodermatitis chronica atrophicans. In endemic regions, outdoor recreational or occupational activities—such as hunting, fishing, camping, mushroom and berry foraging, forestry, farming, and military training—substantially increase exposure risk [7].

In Europe, 65,000–85,000 LB cases are diagnosed and reported annually, with an incidence of 22/ 100,000 person-years in Western Europe [8]. Incidence has risen in recent decades, with geographic expansion into previously non-endemic areas [8]. LB in Italy requires mandatory notification; however, the true incidence is unknown due to inconsistent regional data [9]. Some cases of LB have been reported in Tuscany [9], Trentino and Alto Adige [10], and additional endemic areas have been identified in Emilia-Romagna [11], Abruzzo [12], and Lombardy [13]. Data are limited particularly in southern regions, where sporadic cases have been reported in Sicily [14], Sardinia [15], Lazio [16], and Calabria [17]. From 2001 to 2005, Italy reported the lowest human LB incidence in Europe, ranging from 0.02 to 0.001 new cases per 100,000 inhabitants [18]. Nevertheless, underestimation is likely, with a study indicating a mean annual incidence of 0.887/100,000 in Veneto from 2015 to 2019 [19].

From 2008 to 2021, 87 LB cases were reported in Emilia-Romagna, yet no studies had previously assessed *B. burgdorferi* s.l. circulation in local tick populations. Rising temperatures, drought, deforestation, and changes in biodiversity highlight the need to focus on environmentally influenced vector-borne diseases. Given the epidemiological gaps and the growing influence of climate change on tick ecology, we investigated the prevalence of *B. burgdorferi* s.l. in ticks collected across Bologna (Emilia-Romagna) from February to November 2024, spanning all four seasons. Our aims were to document tick presence in rural and peri-urban environments and to estimate pathogen prevalence, thereby supporting improved awareness and surveillance of LB risks within a One Health perspective.

## 2 Materials and methods

### 2.1 Study areas

**Fig. 1:**
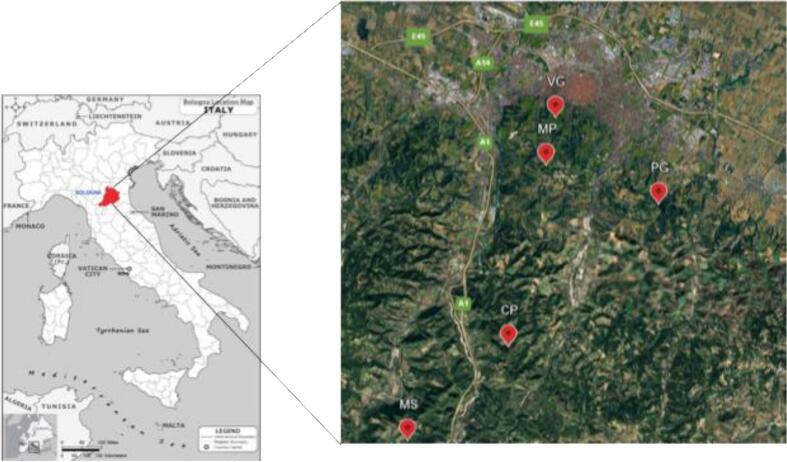
Geolocation of the study area on a geographical map of the municipality of Bologna, illustrating the environmental characteristics of the four sampling sites with *Borrelia burgdorferi* s.l.*-*positive pools of ticks (Supplementary Table 1) (Google Earth, (2025) [Bologna]. Retrieved from https://earth.google.com. Accessed [June2025]). Geolocation of the study area on a geographical map of the municipality of Bologna, illustrating the environmental characteristics of the four sampling sites with *Borrelia burgdorferi* s.l.*-*positive pools of ticks (Supplementary Table 1) (Google Earth, (2025) [Bologna]. Retrieved from https://earth.google.com. Accessed [June2025]).

The study was conducted in the Emilia-Romagna region, located in northeastern Italy, extending from the Adriatic Sea (east) to Apennines (west and south). Its extensive forested areas, vegetation-rich mountain landscapes, and warm, humid climate create highly suitable ecological conditions for the survival and spread of ticks and tick-borne pathogens. The study areas covered Bologna, a territory intersected by the Reno and Savena rivers and situated at the foothills of the Apennines, characterized by numerous forests, peri-urban green spaces, and public parks. A total of twenty-four sites were sampled, each represented by five sites across the Metropolitan City of Bologna (Table 1). These categories span a spectrum from very high to minimal disturbance, based on how closely each site aligns with ecological “naturalness” criteria. School gardens fell at the very high end of the scale, while public parks and urban green areas were classified as high disturbance. Medium and low disturbance levels captured partially unmanaged areas and sites with connections to wild habitats, distinguished by how many of the naturalness criteria they satisfied. At the opposite end of the spectrum, protected areas and ecological restoration zones—such as forested regions and grassland habitats that host diverse wildlife communities, including wolves, wild boar, roe deer, and numerous bird species—were classified as minimally disturbed (Fig. 1, Supplementary Table 1).

### 2.2 Questing ticks

The study was conducted across four seasons, with the initial sampling date on February 2 and the final date on November 4, 2024. A total of 24 locations were sampled, comprising 59 points. Each point was revisited once per season, resulting in four visits throughout 2024. At each site, a 25 m^2^ area was sampled using a 1 m^2^ white flannel blanket dragged along the vegetation with five 5-m swipes, covering the diagonal and four sides. Collected ticks were stored in plastic tubes containing grass and a water-soaked cotton ball.

**Table 1:** Diversity of ticks collected during the study period categorized by season, developmental stage, and pooling strategy.

Season	Total number of ticks	Number of samples	Number of larvae (pools)	Number of nymphs (pools)	Number of adults	Identified species of nymphs (pools)	Identified species of adults
Winter	156	27	5 (2)	146 (20)	5	*Ixodes ricinus* (20)	*Ixodes ricinus* (5)
Spring	240	48	76 (7)	148 (25)	16	*Ixodes ricinus* (24) *Rhipicephalus* sp. (1)	*Ixodes ricinus* (9) *Rhipicephalus* sp. (3) *Dermacentor* sp. (2) *Hyalomma* sp. (2)
Summer	396	37	367 (30)	27 (5)	2	*Ixodes ricinus* (5)	*Ixodes ricinus* (1) *Dermacentor* sp. (1)
Autumn	95	18	62 (8)	31 (18)	2	*Ixodes ricinus* (30)	*Ixodes ricinus* (2)

### 2.3 DNA extraction

All ticks were identified by morphology under a stereomicroscope [20], [21]. After identification by species, developmental stage, and sex, the ticks were grouped by site and life stage with a maximum of 15 ticks per pool and stored at +4 °C, except for adults, which were analyzed individually. DNA extraction involved washing in ethanol and distilled water, followed by cutting ticks into sections and homogenizing with a Tissue Lyser II (Qiagen, Hilden, Germany). The tick mash was resuspended in lysis buffer and Proteinase K contained in the extraction kit TANBead® Nucleic Acid Extraction Kit (Taiwan Advanced Nanotech Inc., Cat# W66PA46-MS,), left overnight at 56 °C and then extracted in the TANBead Maelstrom 9600 Nucleic Acid Extractor. DNA was later quantified with the Qubit™ dsDNA High Sensitivity Assay Kit (Thermo Fisher Scientific, Waltham, MA, USA, Cat# Q32851).

### 2.4 Identification of B. burgdorferi s.l.

The extracted DNAs were screened for *B. burgdorferi* s.l. by real-time PCR targeting the 23S rRNA gene as described by Mysterud et al. [22] using the forward primer CGAGTCTTAAAAGGGCGATTTAGT, the reverse primer GCTTCAGCCTGGCCATAAATAG and [FAM]AGATGTGGTAGACCCGAAGCCGAGTG[BHQ1] probe. We used as a positive control DNA from *Borrelia burgdorferi* sensu stricto strain isolated from an infected tick, while nuclease-free water served as the negative control. The infection rate of *B. burgdorferi* s.l. was estimated using maximum likelihood estimate of prevalence (MLE) calculated only for nymphs pools. Prevalences were estimated assuming perfect sensitivity and specificity of pathogen detection using the EpiTools Epidemiological Calculator “Pooled prevalence for variable pool size and perfect tests” Method 7 (AusVet Animal Health Service, https://epitools.ausvet.com.au/userguideseven) with corresponding 95% confidence intervals [23], [24]. Calculations were performed separately for nymphs and adults, provided that the number of sampled ticks was greater than zero. Differences in seasonal pool positivity rates were assessed using Fisher's exact test, based on the number of PCR-positive and PCR-negative pools per season.

### 2.5 Real-time microfluidic PCR

Samples tested positive by real-time PCR were reanalysed by microfluidic real-time PCR using the Biomark X9™, which allows high-throughput detection of numerous targets simultaneously, consuming only a few microliters of sample and reagents [25]. For this purpose, we used a 48.48 Dynamic Array™ (StandardBiotools, France, Cat# BMK-M-48.48GT), a chip that includes two sets of 48 inlets, allowing loading of 48 samples and 48 PCR assay mixtures, TaqMan Gene Expression Master mix (Applied Biosystems, Italy, Cat# 4369016) and specific primers and TaqMan probes (*Borrelia* spp.*, B. lusitaniae, B. spielmanii, B. valaisiana, B. afzelii, B. garinii, B. miyamotoi, B. burgdorferi s.s.*) (Supplementary Table 2). Before this microfluidic real-time PCR, samples were subjected to a PCR pre-amplification step using the Preamp Master Mix (Standard BioTools, France). This step increased the sensitivity of the microfluidic PCR for pathogen detection [26].

## 3 Results

### 3.1 Tick collection

Between February and November 2024, a total of 887 ticks were collected, with the highest numbers recorded in spring (*n* = 312) and autumn (*n* = 278), followed by summer (*n* = 197) and winter (*n* = 100). Ticks were grouped into 130 samples based on developmental stage and sampling site (Table 1). The majority of ticks were identified as *I. ricinus* (91%), followed by *Rhipicephalus* spp. (4%), *Dermacentor* spp. (3%), and *Hyalomma* spp. (2%).

The site with the highest number of ticks was Parco Storico di Montesole (minimal anthropogenic disturbance), with a total of 570 ticks (253 ticks per 100 m^2^). This was followed by Contrafforte Pliocenico (66 ticks per 100 m^2^), Parco di Villa Ghigi (64 ticks per 100 m^2^), Parco di Monte Paderno (56 ticks per 100 m^2^), and Parco dei Gessi (46 ticks per 100 m^2^) (Supplementary Table 1, Fig. 1). Of the 130 pools, 47 (36%) were composed of larvae, 58 (45%) of nymphs, and 25 (19%) were adult ticks, which were analyzed individually.

### 3.2 Prevalence of B. burgdorferi s.l.

A total of 96 *Ixodes ricinus* samples were screened for *B. burgdorferi* s.l. DNA using real-time PCR, yielding 30 positives—28 pooled nymphs and 2 adult ticks (Table 2). Although 130 tick samples were collected, only the *I. ricinus* samples (96/130)—the competent vector of *B. burgdorferi* s.l. [27]—were included in the analysis, while the remaining non-*Ixodidae* samples, all negative, were excluded from prevalence calculations. The cycle threshold (Ct) values of the positive samples ranged from 25 to 39, with a mean Ct value of 34. Nymphs exhibited the highest positivity rate, with 28 positive pools out of 58 (48%), followed by adults with a positivity rate of 8% (2/25). In contrast, all larvae tested negative (Supplementary Table 1, Table 2). All samples tested positive for the presence of *B. burgdorferi* s.l. DNA were identified as *I. ricinus* ticks.

The estimated prevalence of *B. burgdorferi* s.l. was 10% in nymphal pools (95% CI 6.6–13.5), and 8% in adults (95% CI 1.4–22.7), as calculated using MLE. A statistically significant difference in tick pool positivity was observed across seasons (Fisher's exact test, *p* = 0.015). Positivity rates ranged from 15% in winter to 60% in summer, with intermediate values in spring (48%) and autumn (56%), based on the proportion of PCR-positive per season.

**Table 2:** Seasonal detection of *B. burgdorferi* s.l. DNA in pools of *I. ricinus* nymphs collected in five sampling sites within the municipality of Bologna. Values represent the Maximum Likelihood Estimation (MLE) of infection rate per 100 nymphs (95% CI). “–” indicates that no ticks were collected or no pools tested positive. Abbreviations: MS = Parco Storico di Montesole; CP = Contrafforte Pliocenico; PG = Parco dei Gessi; MP = Parco di Monte Paderno; VG = Parco di Villa Ghigi.

	Number of collected ticks	Number of positive pools	Maximum Likelihood Infection Rate (95% CI)*					
			**MS**	**CP**	**PG**	**MP**	**VG**	**Total**
Winter	146	3	–	–	89% (65.9–98.8)	97% (39.6–100)	–	2.2% (0.5–5.6)
Spring	148	12	9.6% (3.5–20,1)	13.1% (4.2–28.9)	20.6% (3.7–52.5)	–	94% (43.9–100)	10.3% (5.6–16.9)
Summer	27	3	16.7% (4.3–39.5)	–	–	–	–	15.6% (4.0–37.2)
Autumn	31	10	47.6% (27–69.2)	–	–	–	–	37.9% (21–57.4)

### 3.3 Identification of B. burgdorferi species

Of the 30 samples tested positive for the presence of *B. burgdorferi* s.l. DNA, 19 DNA extracts were analyzed for species identification within the *B. burgdorferi* s.l. complex using high-throughput real-time microfluidic PCR. The remaining 11 samples were not tested due to insufficient volume for further analysis. All 19 tested samples originated from nymph pools of *I. ricinus*. The method provided interpretable results in eight out of 19 cases, limited by the high Ct values and limited sample volume. Of the eight positive samples, four tested positive for *Borrelia* spp., indicating the presence of a bacterium of the *Borrelia* genus not included in the detection panel used. *Borrelia afzelii*, *B. lusitaniae*, and *B. garinii* were identified in nymph pools collected from the Parco Storico di Montesole during spring, summer, and autumn, respectively (Table 3).

**Table 3:** Identification of different *Borrelia burgdorferi* sensu lato genospecies in PCR-positive pools of *I. ricinus* nymphs detected through high-throughput real-time microfluidic PCR. Each pool identification number corresponds to a unique nymph pool collected in the study area. The table reports only pools that tested positive, indicating the number of nymphs per pool, sampling site, and season. The designation *Borrelia* sp. indicates detection at the genus level when none of the species-specific targets included in the panel (*B. afzelii*, *B. garinii*, *B. lusitaniae*, *B. spielmanii*, *B. miyamotoi*) amplified. Abbreviations: MS = Parco Storico di Montesole; PG = Parco dei Gessi.

**Pool identification number**	**Ticks count per pool**	**Sampling site**	**Season**	***B. burgdorferi* s.l. high-throughput real-time microfluidic PCR-based detection**
7	3	MS	Autumn	*B. garinii*
20	2	MS	Autumn	*B. garinii*
32	6	PG	Spring	*Borrelia* sp.
37	7	PG	Spring	*Borrelia* sp.
62	2	MS	Spring	*Borrelia* sp.
63	2	MS	Spring	*B. afzelii*
76	5	MS	Summer	*Borrelia* sp.
91	3	MS	Summer	*B. lusitaniae*

## 4 Discussion

The prevalence of *B. burgdorferi* s.l. infection in questing ticks is a critical factor in assessing the risk of Lyme borreliosis (LB). However, prevalence data remain fragmented worldwide, both in ticks and in human and animal hosts, resulting in substantial knowledge gaps and a likely underestimation of true infection rates. This is especially true in Italy, where epidemiological data on LB are limited and surveillance efforts are sporadic. Inconsistent reporting practices further hinder accurate assessments, underscoring the urgent need for strengthened surveillance. The observed seasonal variation in tick pool positivity suggests that *B. burgdorferi* s.l. circulation within *I. ricinus* populations is influenced by ecological and biological factors that fluctuate over the year. Seasonal differences in tick activity, host interactions, and environmental conditions are known to shape pathogen transmission dynamics, and the pattern observed in this study aligns with these broader ecological processes. While the specific drivers cannot be determined from our data alone, the overall trend is consistent with established seasonal patterns reported for *I. ricinus* and associated pathogens [4]. Overall, the data indicate that pathogen circulation is not restricted to traditionally recognized high-risk months and that both spring and autumn represent critical periods for *B. burgdorferi s*.l. transmission in the Bologna area.

Seasonal studies conducted in various Italian regions during spring and summer report varying prevalences of *B. burgdorferi* s.l. in ticks, including 20% in Friuli Venezia Giulia [28], 8.5% in Tuscany [29], 26.7% in Emilia-Romagna [30], 36% in Lazio [31], and 10% in Piedmont [32].

Despite the limited sample size for species identification, *B. afzelii* and *B. garinii* were confirmed as circulating genospecies in Italy, consistent with their known role as causative agents of LB in Europe [27]. *Borrelia burgdorferi* s.s. was not detected in our study, although it has been identified in ticks from other regions in Italy [33] and is more prevalent in North America [34]. Tick density is another crucial variable influencing the risk of LB. Forest habitats with minimal anthropogenic disturbance typically harbour the highest tick densities, sometimes exceeding 100 ticks per 100 m^2^. For instance, in the Parco Storico di Montesole, a protected area distant from human settlements and rich in wildlife, tick density reached 253 ticks per 100 m^2^. Forested areas such as Parco dei Gessi also supported multiple tick species and high tick densities, reinforcing the role of these habitats as key environments for tick survival and activity [34].

Furthermore, infected tick density -the number of *B. burgdorferi* s.l.-positive ticks per unit area- may correlate better with human LB incidence than prevalence alone [34]. The spatial distribution of *B. burgdorferi* s.l. positivity across seasons revealed notable heterogeneity. In winter, only two out of twenty-four sampling sites tested positive (MLE 2.2%), both characterized by low but not minimal anthropogenic disturbance. In contrast, spring showed broader spatial circulation, with four positive sites (MLE 10.3%), including three with minimal and one with low disturbance. Despite higher MLE values in summer (15.6%) and autumn (37.9%), only a single site with minimal anthropogenic impact tested positive in each of these seasons. These patterns suggest that while infection intensity may peak in localized foci during warmer months, broader spatial dissemination may occur in spring. Notably, the Parco Storico di Montesole not only had the highest tick density but also yielded the majority of *B. burgdorferi* s.l.-positive samples in three out of four seasons (excluding winter) and represented the largest sampling area, indicating its potential role as a persistent ecological reservoir [7]. Importantly, high tick density and high *B. burgdorferi* s.l. prevalence do not necessarily translate into elevated human case numbers, as exposure risk is strongly modulated by human behaviour, frequency of visits to tick habitats, and awareness of preventive measures.

In conclusion, this study provides the first year-round molecular surveillance of *B. burgdorferi* s.l. in questing ticks within the municipality of Bologna, revealing marked seasonal variation in both tick abundance and infection rates. By demonstrating that spring and autumn carry the highest risk of *Borrelia* transmission and by identifying key genospecies such as *B. afzelii* and *B. garinii* in local tick populations, our findings contribute essential data to the limited epidemiological landscape of LB in Italy [9], [19].

From a One Health perspective, these results highlight how pathogen circulation emerges from the interplay between environmental conditions, wildlife host communities, and human land use. Seasonal peaks in infection reflect not only tick phenology but also fluctuations in reservoir host availability and climatic suitability, emphasizing that *B. burgdorferi* s.l. transmission is tightly coupled to ecosystem dynamics [2], [4]. The strong association between low-disturbance forested habitats and high tick density underscores the role of landscape structure and biodiversity in shaping vector ecology [34]. Conversely, the detection of infected ticks in peri-urban and recreational areas illustrates how anthropogenic disturbance can bring humans, domestic animals, and wildlife into closer contact, increasing opportunities for spillover [31]. These findings reinforce the need for integrated surveillance systems that combine entomological monitoring, wildlife host assessments, environmental data, and human case reporting. Prevention strategies should extend beyond individual protective measures and include targeted habitat management and public education aligned with seasonal risk. Coordinated action across human health, veterinary medicine, and environmental management is essential to address the complex socio-ecological drivers of Lyme borreliosis in Bologna. Future research integrating climatic modelling, host community dynamics, and human exposure patterns will be crucial to identify hotspots of infected tick density and support more effective interventions.

The following are the supplementary data related to this article.

Supplementary Table 1

Supplementary Table 2

## CRediT authorship contribution statement

**Alessandra Mistral De Pascali:** Validation, Methodology, Investigation, Formal analysis, Data curation, Conceptualization. **Laura Dionisi:** Writing – original draft, Investigation, Formal analysis, Conceptualization. **Bianca Vandelli:** Methodology, Investigation, Conceptualization. **Carola Valenti:** Methodology, Formal analysis, Conceptualization. **Sara Rigamonti:** Investigation. **Emanuela Olivieri:** Investigation. **Nadia Vicari:** Investigation. **Martina Brandolini:** Formal analysis. **Ludovica Ingletto:** Formal analysis. **Giulia Gatti:** Visualization. **Claudia Colosimo:** Validation. **Massimiliano Guerra:** Investigation. **Giorgio Dirani:** Investigation. **Silvia Zannoli:** Investigation. **Laura Grumiro:** Supervision. **Sofia Montanari:** Visualization. **Anna Marzucco:** Validation. **Monica Cricca:** Writing – review & editing, Supervision. **Roberto Cazzolla Gatti:** Writing – review & editing, Supervision. **Alessandra Scagliarini:** Writing – review & editing, Supervision. **Vittorio Sambri:** Writing – review & editing, Validation, Supervision, Project administration, Funding acquisition.

## Funding statement

This work was supported by 10.13039/501100000780EU funding within the MUR PNRR Extended Partnership initiative on Emerging Infectious Diseases (Project no. PE00000007, INF-ACT).

## Declaration of competing interest

The authors declare that they have no known competing financial interests or personal relationships that could have appeared to influence the work reported in this paper.

## Data Availability

No data was used for the research described in the article.
